# The promise of stem cells in the therapy of Alzheimer’s disease

**DOI:** 10.1186/s40035-015-0029-x

**Published:** 2015-04-28

**Authors:** Chunmei Yue, Naihe Jing

**Affiliations:** State Key Laboratory of Cell Biology, Institute of Biochemistry and Cell Biology, Shanghai Institutes for Biological Sciences, Chinese Academy of Sciences, Shanghai, 200031 China

**Keywords:** Alzheimer’s disease, Stem cell-based therapy, Basal forebrain cholinergic neurons, Cognitive impairment, Embryonic stem cells

## Abstract

Alzheimer’s disease (AD), a common neurodegenerative disorder associated with gradually to dramatic neuronal death, synaptic loss and dementia, is considered to be one of the most obscure and intractable brain disorders in medicine. Currently, there is no therapy clinically available to induce marked symptomatic relief in AD patients. In recent years, the proof-of-concept studies using stem cell-based approaches in transgenic AD animal models provide new hope to develop stem cell-based therapies for the effective treatment of AD. The degeneration of basal forebrain cholinergic neurons (BFCNs) and the resultant cholinergic abnormalities in the brain contribute substantially to the cognitive decline of AD patients. The approches using stem cell-derived BFCNs as donor cells need to be developed, and to provide proof of principle that this subtype-specific neurons can induce functional recovery of AD animal models. With the continuous scientific advances in both academic and industrial fields, the potentials of stem cells in cellular neuroprotection and cell replacement *in vivo* have been elucidated, and stem cell-based therapy for repairing degenerative brains of AD is promising.

Alzheimer’s disease (AD), a multifactorial syndrome, is believed to be the most common form of neurodegenerative dementia. Previous studies have demonstrated that AD is associated with several distinct neuropathological features, mainly as extracellular Aβ plaques, intracellular neurofibrillary tangles and neuron lost. Currently, there is no effective therapies available for the treatment of AD, and the widely used medicine, including cholinesterase inhibitors, have very modest clinical effects in treating the symptoms of AD, such as language loss, cognitive deficits and behavioral impairments. It was concluded that AD epitomized the mechanistic ignorance and therapeutic nihilism that pervaded the study of neurodegeneration in humans [1]. In 2000, approximately 4.5 million people suffered from AD in the America, which is expected to 13.2 million by the year 2050 [2]. The AD patients by no means will become one of the largest public health challenges and place heavy demands on the health care system.

The stem cells hold potentials to develop cell replacement therapies for various neurodegerative disease, including AD. The subtype-specific neurons from stem cells might be the most ideal donor cells to replace the same type of neurons lost through disease. The embryo stem cell (ESC)-derived dopaminergic neurons were shown to correct functional deficits in animal models of Parkinson’s disease [3,4]. The attempts to develop stem cell-based therapies for AD have used several types of cells in AD animal models, such as adult neural stem cells [5], ESC-derived neural precursor cells [6], a couple of mesenchymal stem cells [7,8], and astrocytes [9]. These studies had demonstrated that transplanted cells partially rescue the cognitive deficits of AD animals. Among the widespread neuron and synaptic loss in AD brain, the degeneration of basal forebrain cholinergic neurons (BFCNs) and severe devastation of basal forebrain cholinergic innervation of cerebral cortex and hippocampus in the brain of AD patients are thought to play essential role in memory impairment, cognitive deficits, and finally lead to dementia [10,11]. The replacement procedures using stem cell-derived BFCNs as donor cells need to be establised, and the functional efficacy of grafted BFCN needs to be determined.

The purpose of this short review is to briefly summarize the continuous advances in the development of stem cell-based approaches for AD in both academic and industrial fields, and carefully predict the promising potentials of stem cells for repairing degenerative brains of AD.

## The scientific advances in academic research

Recently, multiple attempts have used different types of stem cells as donor cells and investigated the effects of stem cell transplantation on relieving neuropathological symptoms and restoring cognitive function of transgenic AD animal models. Both cultured adult and neonatal mouse astrocytes are transplanted into the hippocampus of ApdE9 AD mice. Seven days later, these astrocytes are found mainly near Aβ deposits and internalize human Aβ immunoreactive material *in vivo*. This study supports the role of astrocytes as active Aβ clearing cells in the brain, which may have important implications for future development of therapeutic strategies for AD [9]. Adult neural stem cells from newborn GFP transgenic mice have been transplanted into the hippocampus of aged 3xTg-AD mice, a model that recapitulates many of the salient features of AD. The neural stem cell transplantation induces a robust enhancement of BDNF-mediated hippocampal synaptic density and rescues the spatial learning and memory deficits of AD mice, without altering Aβ deposits. This study suggests that modulation of neurotrophin levels could provide a viable approach in the development of stem cell-based therapies to treat AD in future [5]. The human umbilical cord blood-derived mesenchymal stem cells are transplanted into hippocampus of AD mice, which rescues memory deficits of host mice by reducing neuronal apoptosis [7]. The bone marrow-derived mesenchymal stem cells are transplanted into hippocampus of AD mice, which reduces Aβ deposits and rescues memory deficits of host mice [8]. The mouse embryonic stem cell-derived cholinergic motor neuron precursors are transplanted into the right NBM of rats with unilateral NBM lesion and memory deficits, and promote the memory recovery of host rats [6]. The human iPSC-derived neuronal precursors from SHH/RA treatment are transplanted into the hippocampus of AD transgenic mice and restore spatial memory of AD mice [12].

In summary, different types of donor cells that are transplanted mainly into hippocampus of AD model animals exhibit distinct effect on neuropathological symptoms relief and cognitive function restoration (Table 1). All these studies suggest the perspective possibility of using stem cells to develop therapeutics for AD.

**Table 1:** **The reported stem cell-based therapies in AD animal models**

**Donor cells**	**AD models**	**Graft site**	**Behavior recovery**	**Possible mechanism**	**Key refs.**
Mouse astrocytes	ApdE9 transgeneic mouse	Hippocampus	Not known	Aβ deposits clearing	[9]
Mouse adult NSCs	3xTg transgeneic mouse	Hippocampus	Learning and memory	BDNF induction	[5]
Mouse cholinergic motor neuron precursors	Rat with NBM lesion	NBM	memory recovery	Not Known	[6]
Human UCB-MSCs	Not known	Hippocampus	Memory	Reducing neuronal apoptosis	[7]
Mouse BM-MSCs	APP/PS1 transgeneic mouse	Hippocampus	Memory	reducing Aβ deposits	([8]
Human iPSC-derived motor neuron precursors	PDAPP transgeneic mouse	Hippocampus	Spatial Memory	Not Known	[12]

NSCs, neural stem cells, NBM, nucleus basalis of Meynert, UCB-MSCs, umbilical cord blood-derived mesenchymal stem cells, BM-MSCs, bone marrow-derived mesenchymal stem cells, PDAPP, PDGF promoter driven amyloid precursor protein transgenic mice.

## The promising application of basal forebrain cholinergic neurons (BFCNs) in stem cell-based treatment of AD

There are two major types of cholinergic neurons in the brain, the projection neurons in the basal forebrain, known as BFCNs, and interneurons in the striatum. In rodent brain, BFCNs originating in the most ventral regions of the developing telencephalon at embryonic day 11, display highly discrete locations and possess striking anatomic heterogeneities [13]. In primate brain, the BFCNs situate in the medial septal nucleus, vertical limb of the diagonal band of Broca and nucleus basalis of Meynert (NBM), and typically innervate all parts of cerebral cortex, hippocampus, entorhinal cortex and amygdala [14]. The BFCNs play essential roles in various aspects of cognitive function, such as learning, memory and attention, and the cholinergic blockade disrupts the cognitive function of normal humans [10,15,16]. Numerous studies show the severe devastation of basal forebrain cholinergic innervation and resultant declined cholinergic neurotransmission in the brains of AD patients, and even in the early stage of AD patients [17–20]. Also, the most severely affected areas in AD brain are within the temporal lobes, especially within the hippocampus and entorhinal cortex [21,22]. These studies point out that the degeneration of BFCNs essentially contribute to the cognitive deficits and pathogenesis of AD, suggesting that BFCNs might be an ideal type of donor cells to ameliorate the cognitive symptoms associated with AD. Even if the hypothesis by using BFCNs as donor cells for treating AD is exciting, none of the studies listed above has attempted to test if BFCNs can restore cholinergic function and alleviate cognitive deficits in AD model animals. Indeed, BFCNs cannot be stably generated from pluripotent stem cells, either mouse or human embryonic stem cells (ESCs) for the transplantation experiments. Up to date, the optimal strategy directing the differentiation of pluripotent stem cells into BFCNs *in vitro* has not been established, which is mainly due to the unclear molecular basis of the differentiation and development of BFCNs *in vivo*. A number of endogenous neurotrophic factors, such as nerve growth factor (NGF), brain-derived neurotrophic factor (BDNF), basic fibroblast growth factor (bFGF) and bone morphogenetic protein 9 (BMP9), have been reported to participate and promote the survival, growth, and differentiation of cholinergic neurons, and probably BFCNs in the brain [23–25]. The various transcription factors, such as Mash1, Islet1, Nkx2.1, Lhx8, and Olig2, act hierarchically to determine the cell fate and function coordinately to maintain the cell identity of striatal cholinergic neurons, but the dynamics expression and genetic determinants of these factors in the development of BFCNs are incompletely understood [26–29].

Due to the unclear elucidation of key developmental factors and extracellular signals that control the development of BFCNs *in vivo*, the successful derivation of BFCNs from mouse ESCs has not been reported, and the studies on differentiation of human ESCs to BFCNs has been sparse. Few years ago, Bissonnette and colleagues [30] reported the derivation of a predominantly pure BFCN population from human ESCs. They used diffusible ligands present in forebrain, including RA in the early stage of neural induction in their method. These human ESC-derived BFCN neurons express typical cholinergic neuronal markers, and stably and functionally engraft in murine ex vivo hippocampal slices through generating electrophysiologically functional cholinergic synapses and firing action potentials. Two years later, Liu and colleagues have developed a method for differentiating hESCs to a nearly homogeneous population of MGE-like progenitors [31]. Following their differentiation protocols, these MGE-like progenitors finally give rise to mixed cell population containing nearly equal amount of GABAergic interneurons and BFCNs. After transplantation into the hippocampus of severe combined immunodeficiency (SCID) mice in which BFCNs and some GABA neurons in the medial septum had been destroyed by muP75-saporin, human MGE-like progenitors produced BFCNs that synaptically connected with endogenous neurons. More excitingly, mice transplanted with MGE-like progenitors showed improvements in learning and memory deficits. The function of ESC-derived BFCNs has been tested from the murine *ex vivo* hippocampal slices to the severe combined immunodeficiency mice with medial septum lesion, indicating that one step has moved forward on the road of exploring the subtype-specific neuron-based cell therapy for AD. Recently, we have successfully differentiated both mouse and human ESCs into BFCNs through a highly pure population of BFCN progenitors. Both mouse and human ESC-derived BFCN progenitors are transplanted into the NBM of transgenic AD model mice, 5XFAD and APP/PS1, and specifically differentiated into mature and functional cholinergic neurons *in vivo*. These exogenous cholinergic neurons exhibited typical basal forebrain cholinergic projection and migration patterns, and morphologically and functionally incorporate into the endogenous projection system. Importantly, AD mice with transplanted BFCN progenitors exhibited improved learning and referenced memory abilities in the behavior test, demonstrating the feasibility of using ESC-derived BFCNs for the development of stem cell therapy for AD (unpublished data).

It is well accepted that the degeneration of BFCNs and resultant devastation cholinergic innervation spread a large area of brain, from basal forebrain to hippocampus and entorhinal cortex in temporal lobe. However, the time that BFCNs start to degenerate through disease processing remains unclear, and mechanism underlying the BFCN degeneration needs to be elucidated. These unknown events make it more difficult to optimize the cell replacement procedures using BFCNs. The optimal transplantation site remains to be determined. The survival, proliferation, cell fate specification and differentiation of grafted BFCN progenitors need to be fine controlled. The biochemical functions, migration, projection and integration of exogenous BFCNs remain largely unknown. Obviously, the procedures of stem cell therapies using ESC-derived BFCNs for the treatment of AD are far from optimal, and the challenges ahead will be tremendous (Table 2).

**Table 2:** **The advantages and disadvantages of using BFCNs as donor cells in the stem cell-based approaches for AD**

**Advantages**	**Disadvantages**
Subtype-specific replacing the lost endogenous BFCNs in basal forebrain	The standard preparation of homogeneous human BFCN progenitors
Repairing the dysfunctions of basal forebrain cholinergic system by releasing acetylcholine, neurotrophic factors or cytokines	The efficient cholinergic specificaiton of BFCN progenitors, and long term survival of cholinergic derivatives *in vivo*
Functionally integrating into the endogenous basal forebrain neural circuitries	The disabilities of lone-distance innervation from the basal forebrain to hippocampus and cortex
Rescuing the innervation track along the host cholinergic projections through out the basal forebrain	The uneven and patchy migration through the basal forebrain to hippocampus and cortex

## The translational perspective in stem-cell based therapy of AD in industry

Even with the continuous progress in developing stem cell-based therapy for AD in academic field, the challenges in the early stage of stem-cell based therapy to treat neurodegeneratice diease, including AD, are obviously insurmountable. However, the transition from proof-of-concept studies in AD animals to human clinical trials in AD patients has already been underway in the industrial field, which comes sooner than one might think [32]. In 1999, based on their groundbreaking finding of cell surface markers for adult human neural stem cell, the scientists of StemCells Inc. have successfully isolated a highly purified, expandable population of neural stem cells from human brain tissue by using monoclonal antibodies against the cell surface markers. Then, the human neural stem cells have been prepared under controlled conditions and cGMP standards and named HuCNS-SC cells. The rigorous preclinical studies have shown that HuCNS-SC cells can survive long-term with no evidence of tumor formation or adverse effects, and engraft, migrate, differentiate into neurons, astrocytes and oligodendrocytes. In 2011, StemCells established collaborations with some famous AD research groups in the world to study the therapeutic potential of HuCNS-SC cells in AD. The preliminary results showed HuCNS-SC cells can survive in brain of AD animal models, and StemCells Inc. hopes to advance toward clinical testing of HuCNS-SC cells in AD patients. Different from StemCell, the NeuralStem has devoted to isolate and expand regionally specific cells. NeuralStem has announced their preclinical study by using engineered human cortex-derived neural stem cells for AD. The neural stem cells can secrete human insulin-like growth factor 1 and rescue spatial learning and memory deficits after transplanted into an animal model of AD.

Although the stem cell-based therapy for AD has commenced, it is still in the earlier stage compared to development of stem cell therapy candidates and technological platforms for other brain or spinal cord disorders. The clinical trial of human ESC-derived oligodendrocyte progenitors from Geron for spinal cord injury has been authorized by FDA, which is a milestone for the stem cell therapy field. The clinical-grade neural stem cell CTX from ReNeuron has been appoved for the phase II clinical trials for treatment of stroke. With the continued progress and mutual involvement of scientific, ethical, regulatory, translational and clinical fields, more and more companies start to involve in and make efforts to develop rational stem cell-based therapies for the treatment of AD.

The progresses in both scientific and industrial fields have provided proof of principle that the stem cells can induce marked improvements in AD animals, and may lead to valuable improvement in clinical trials. But, there are still different kinds of challenges ahead before stem cell-based therapy can move to the clinic as a treatment for AD: a) the standard preparation of cells suitable for transplantation has not been achieved, b) the present cell replacement procedures are far from optimal, c) it is still unclear the mechanism underlying symptomatic relief upon cell transplantation, d) it still needs to be determined the chronic inflammatory and immune response due to the cell grafts. Even though, the continued understanding in cell biology of donor cells, the advances in stem cell transplantation and involvement of clinical activities will facilitate the development of stem cell-based therapeutic strategies for AD, and the perspective of stem cell therapy for the treatment of AD is expected.
